# Extracellular Vesicles From Pathological Microenvironment Induce Endothelial Cell Transformation and Abnormal Angiogenesis via Modulation of TRPV4 Channels

**DOI:** 10.3389/fcell.2019.00344

**Published:** 2019-12-17

**Authors:** Brianna D. Guarino, Ravi K. Adapala, Anantha K. Kanugula, Nina M. Lenkey, Julie A. Dougherty, Sailaja Paruchuri, Mahmood Khan, Charles K. Thodeti

**Affiliations:** ^1^Department of Integrative Medical Sciences, College of Medicine, Northeast Ohio Medical University, Rootstown, OH, United States; ^2^School of Biomedical Sciences, Kent State University, Kent, OH, United States; ^3^Dorothy M. Davis Heart and Lung Research Institute, The Ohio State University Wexner Medical Center, Columbus, OH, United States; ^4^Department of Emergency Medicine, The Ohio State University Wexner Medical Center, Columbus, OH, United States; ^5^Department of Chemistry, The University of Akron, Akron, OH, United States; ^6^Department of Physiology and Cell Biology, The Ohio State University Wexner Medical Center, Columbus, OH, United States

**Keywords:** angiogenesis, endothelial cells, extracellular vesicles, tumor, TRPV4

## Abstract

The soluble and mechanical microenvironment surrounding endothelial cells influences and instructs them to form new blood vessels. The cells in the pathological tumor microenvironment release extracellular vesicles (EVs) for paracrine signaling. EVs have been shown to induce angiogenesis by communicating with endothelial cells, but the underlying molecular mechanisms are not well known. We have recently shown that the mechanosensitive ion channel transient receptor vanilloid 4 (TRPV4) expression and activity is significantly reduced in tumor endothelial cells (TEC), and that activation of TRPV4 normalized the tumor vasculature and improved cancer therapy. However, whether and how the tumor microenvironment downregulates TRPV4 and transforms the normal endothelial cell phenotype remains unknown. To explore this, we exposed normal human endothelial cells (hNEC) to human lung tumor cell conditioned media (TCM) and measured phenotypic changes and angiogenesis. We found that treatment with TCM transformed hNEC to a TEC-like phenotype (hTEC) as evidenced by increased expression of tumor endothelial cell marker 8 (TEM8) and exhibition of abnormal angiogenesis on 2D-Matrigels compared to normal hNEC. Mechanistically, expression and activity of TRPV4 was decreased in hTEC. Further, when pre-treated with exosome inhibitor GW4869, TCM failed to induce hNEC transformation to hTEC. Finally, addition of purified EVs from TCM induced transformation of hNEC to hTEC as evidenced by abnormal angiogenesis *in vitro*. Taken together, our results suggest that the pathological (tumor) microenvironment transforms normal endothelial cells into a tumor endothelial cell-like phenotype through EVs via the downregulation of TRPV4.

## Introduction

Angiogenesis, the formation of new blood vessels from the existing vessels, is a natural physiological process that occurs in response to tissue oxygen demand. Uncontrolled or insufficient angiogenesis can lead to ischemic heart disease, retinopathy and cancer (Folkman, 2002, 2006; Al-Latayfeh et al., 2012). In fact, solid tumors require angiogenesis for their growth and maintenance; however, tumor angiogenesis is unregulated resulting in aberrant vascular growth i.e., pathological angiogenesis (Jain, 2005a; Jain and Carmeliet, 2012). Therefore, the tumor vasculature exhibits high tortuosity, poor pericyte coverage, abnormal extracellular matrix (ECM) and hyper-permeability, causing an inefficient delivery of chemotherapies to the tumor (Siemann, 2011). Conventional anti-angiogenic therapies targeting soluble factors like VEGF have emerged as a means of overcoming the challenges posed by abnormal tumor vessels. Despite the initial results, which were promising, these anti-angiogenic therapies are often met with new challenges, such as acquired drug resistance by tumor endothelial cells (TEC) (Casanovas et al., 2005). Therefore, alternative approaches, such as restoring adequate blood flow and function of the tumor vasculature, i.e., vascular normalization is an attractive avenue of exploration (Jain, 2005b; Carmeliet and Jain, 2011).

To initiate angiogenesis, tumor cells must communicate with and recruit different cell types to form the tumor microenvironment (TME) which is made up of stromal cells such as pericytes, fibroblasts, endothelial cells (EC), ECM, and immune cells (Nyberg et al., 2008; Watnick, 2012; Samples et al., 2013; Yuan, 2016). These varying cell types release extracellular vesicles (EVs), such as exosomes and microvesicles, into the TME that are then taken up by recipient cells via endocytosis (Han et al., 2019; Maacha et al., 2019). EVs are small vesicles (30–150 nm for exosomes, 100 nm–1 μm for microvesicles) containing nucleic acids and proteins and are an important element of cell-to-cell communication. Emerging evidence has shown that EVs play a role in promoting tumor angiogenesis (Ludwig and Whiteside, 2018; Zimta et al., 2019), however, exact mechanisms have yet to be determined.

We have previously shown that transient receptor potential vanilloid 4 (TRPV4) is downregulated in TEC, and that pharmacological activation of TRPV4 channels normalizes the tumor vasculature and improves cancer therapy (Adapala et al., 2016). However, whether and how the TME induces a downregulation of TRPV4 channels in TEC remains unknown. Therefore, the main goal of this study is to show that tumor cell conditioned media (TCM) can induce downregulation of TRPV4 channels. In the present study, we investigated if a pathological microenvironment, such as TME, can transform normal ECs into a TEC-like phenotype and its underlying mechanisms.

## Materials and Methods

### Tumor Cell Conditioned Media (TCM)

Adenocarcinomic human alveolar basal epithelial cells (A549) were from ATCC and cultured in normal DMEM high glucose media supplemented with 10% fetal bovine serum (FBS) and 1% penicillin-streptomycin. After the cells reached 80% confluence, complete media was replaced with serum free DMEM high glucose for 24 h, collected, and centrifuged at 1200 rpm. After centrifugation, TCM was filtered and stored at −80°C for future use. For the inhibition studies, A549 cells were pre-treated with the exosome inhibitor, GW4869 (10 μM), for 24 h, prior to adding serum free DMEM high glucose as described above.

### Cell Culture

Human microvascular endothelial cells (HMEC-1) were purchased from ATCC (Manassas, VA, United States), cultured in MCDB-131 media supplemented with 10% (FBS), 1% penicillin streptomycin, 1% L-glutamine, 1 μg/mL hydrocortisone, and 10 ng/mL human vascular endothelial growth factor (Kanugula et al., 2019). HMEC-1 were denoted as human normal endothelial cells (hNEC). To investigate the effect of TCM on endothelial cells, hNEC were cultured in a medium containing TCM: HMEC-1 media (75:25) for five continuous passages. At the end of the five passages, these cells exhibited a tumor-endothelial like phenotype and termed as human tumor endothelial-like cells (hTEC).

### Quantitative PCR (qPCR)

RNA was extracted from EC by using the RNeasy Mini Kit (Qiagen, Hilden, Germany) and quantified with a Biotech 96-well plate reader. RevertAid First Strand cDNA Synthesis Kit was used to synthesize cDNA, and Fast SYBR green master mix was used for qPCR on the Fast-Real-Time PCR system (both from Thermo Fisher Scientific). The following real-time primers were obtained from Integrated DNA Technologies (Coralville, IA, United States): β-actin (forward-5′-ACGTTGCTATCCAGGCTGTG-3′, reverse: 5′-GAGGGCATACCCCTCGT-AGA-3′) and TEM8 (forward-5′-GCTATTATGTGTCCCGTCTCTATG, reverse: 5′-GGTGGGTGTTGGAGAGTATTG). Gene expression was normalized to β-actin, and ΔΔC*_*t*_* values were expressed as a fold change relative to hNEC.

### *In vitro* Angiogenesis Assay

Growth factor reduced Matrigel^®^ (BD Biosciences) was placed in a 48-well plate and kept at 37°C for a total of 30 min (Adapala et al., 2016; Thoppil et al., 2016). Cells (1 × 10^5^ cells/well) were plated on the Matrigel and kept at 37°C for 24 h. In some experiments, cells were pre-treated and plated together with Rho kinase inhibitor, Y27632 (10 μM) on Matrigel. Tube length was quantified using ImageJ software. For EV experiments, hNEC cultured in serum free MCDB-131 media combined with normal HMEC-1 media (75:25) were treated with 100 μg/mL of purified EVs (total EV protein) or PBS as control for 48 h before plating them on Matrigel.

### Western Blot Analysis

Cells were lysed in RIPA buffer containing protease and phosphatase inhibitor cocktails (MilliporeSigma and Roche, Basel, Switzerland). Lysates were loaded into 7.5% precast polyacrylamide gels (Bio-Rad) for electrophoresis. Gels were transferred onto a PVDF membrane and blocked in 5% milk powder in tris-buffered saline (TBS) with 0.1% Tween-20. Membranes were incubated overnight at 4°C with primary antibodies: TRPV4 (1:300; Alomone Labs, Jerusalem, Israel, or 1:300; Biorbyt, San Francisco, CA, United States), and GAPDH (1:5000; Cell Signaling Technology). After incubation, membranes were washed 3× with TBS-Tween-20 for 10 min each, followed by 1 h incubation at room temperature in appropriate secondary antibody, goat anti rabbit (1:5000) conjugated with horseradish peroxidase (Cell Signaling Technology). Signals were detected with Clarity western luminol/enhancer solution and peroxide solution (Bio-Rad laboratories, Hercules CA, United States), and developed with a FluorChem M Simple Imager (Protein Simple, San Jose, CA, United States). Quantification was performed using ImageJ software.

### Calcium Imaging

Endothelial cells were cultured on MatTek glass bottom dishes (MatTek, Ashland, MA, United States). Cells were loaded with Fluo-4/AM (4 μM) for 25 min and calcium influx was monitored as previously described (Adapala et al., 2011, 2016) on Olympus FluoView 300 confocal microscope (Olympus, Shinjuku, Tokyo, Japan) after stimulation with the TRPV4 agonist, GSK1016790A (100 nM).

### Immunocytochemistry

Cells were cultured on glass coverslips in a 6-well plate and fixed in 4% paraformaldehyde (PFA) for 20 min. After fixing, cells were washed 3× with 1× PBS, permeabilized for 15 min with 0.25% TritonX-100 solution and blocked for 30 min in 10% FBS-containing media. Cells were incubated for 1 h at room temperature with VEGFR2 primary antibody (1:200; Cell Signaling Technology), washed 3× in 1× PBS, incubated for 1 h at room temperature with appropriate Alexa Fluor conjugated secondary antibody (1:200; Thermo Fisher Scientific). Cells were then washed 3× in 1× PBS and mounted with DAPI containing mounting medium (Vector Laboratories, Burlingame, CA, United States) on glass slides. Images were captured using an Olympus IX-71 fluorescence microscope (Olympus).

### Extracellular Vesicle Isolation and Characterization

Extracellular vesicles were isolated and characterized as previously described (Dougherty et al., 2018). Briefly, ^1^/5 volume of ExoQuick-TC reagent (SBI, Mountain View, CA, United States) was added to the TCM. TCM was then incubated overnight at 4°C, followed by centrifugation at 1,500 × *g* for 30 min (4°C) to pellet EVs. Another round of centrifugation was performed to remove any residue. EVs were re-suspended in PBS and stored at −80°C for future use. Nanoparticle Tracking Analysis (NTA) was performed by diluting EVs with PBS and loading onto a Malvern Nanosight NS300 (Malvern, United Kingdom). Video was analyzed via Nanosight NS300 NTA software v3.00 (Malvern, United Kingdom). EVs were sonicated in ethanol, deposited on 400 carbon coated mesh grids and the cryo-Transmission electron microscopy (TEM) imaging was carried out in a FEI Tecnai F20 microscope operated as described previously (Gao et al., 2014).

### Statistical Analysis

The data was analyzed with student’s *t*-test using SPSS V. 24 software. The significance was set at ^∗^*p* ≤ 0.05; ^****^p ≤ 0.0001. All values expressed as mean ± SEM.

## Results

### Tumor Cell Conditioned Media Transforms Human Normal Endothelial Cells (hNEC) Into a Tumor Endothelial Cell-Like (hTEC) Phenotype

**FIGURE 1:**
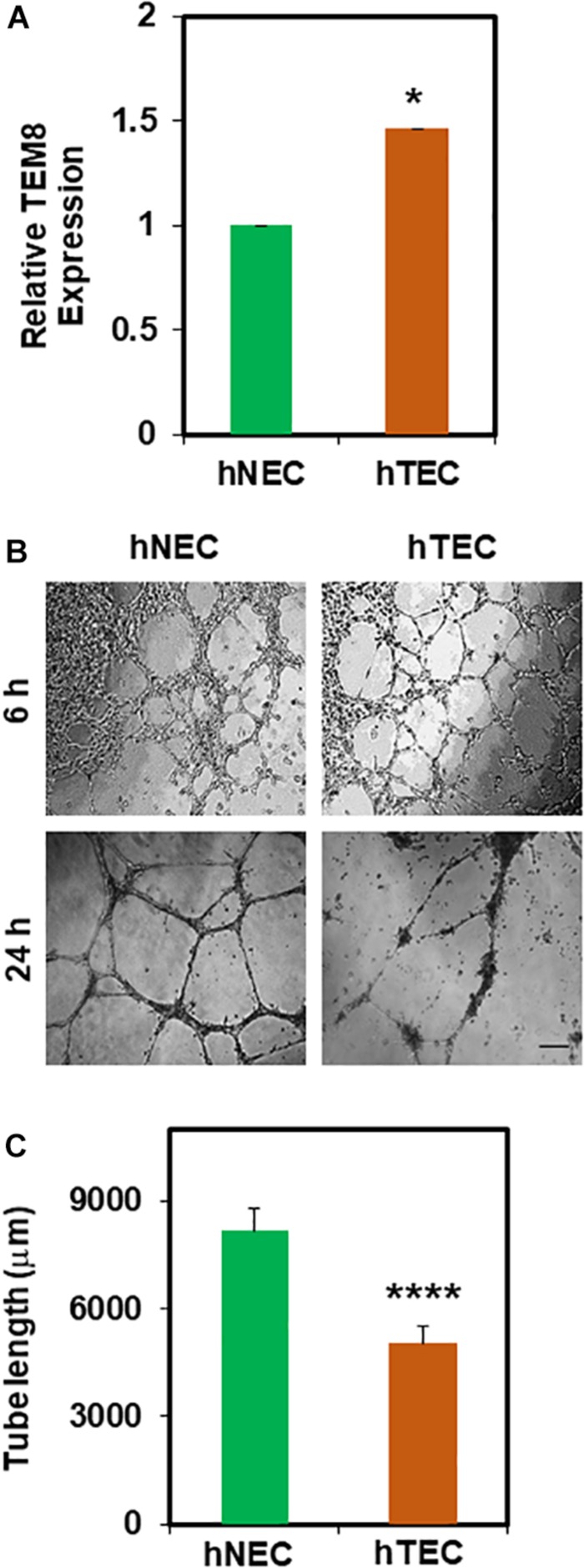
Tumor cell conditioned media (TCM) induces transformation of hNEC to tumor endothelial-like (hTEC) phenotype. **(A)** qPCR analysis showing relative TEM8 gene expression in untreated hNEC and TCM-treated hNEC (hTEC). Note, TEM8 mRNA expression is increased significantly in hTEC (^∗^*p* ≤ 0.05). Gene expression was normalized to β-actin and calculated as relative expression to hNEC. **(B)** Phase contrast images (4×) showing the angiogenic behavior of hNEC and hTEC when plated on 2D Matrigel at high density (100,000/well) at 6 and 24 h. Scale bar = 200 μm. **(C)** Quantitative analysis showing a significant decrease (^****^p ≤ 0.0001) in tube length in hTEC compared to hNEC. The results shown are a mean ± SEM from three independent experiments.

In order to mimic the TME *in vitro*, we repeatedly exposed hNEC to TCM. First, we found an increased gene expression of tumor endothelial marker 8 (TEM8) in TCM-treated hNEC (hTEC) compared to untreated cells (Figure 1A). Next, in *2D-* angiogenesis assays, while hNEC formed robust tubes that stabilized until 24 h, TCM-treated EC formed tubes at 6 h but were collapsed and retracted at 24 h (Figures 1B,C; *p* ≤ 0.0001), which is reminiscent of mouse TEC (Adapala et al., 2016). Taken together, these findings suggest that TCM can transform normal EC (hNEC) into a tumor endothelial cell-like (hTEC) phenotype.

### Tumor Cell Conditioned Media Induces hNEC Transformation to hTEC via Downregulation of TRPV4 and Reduction of Perinuclear VEGFR2

**FIGURE 2:**
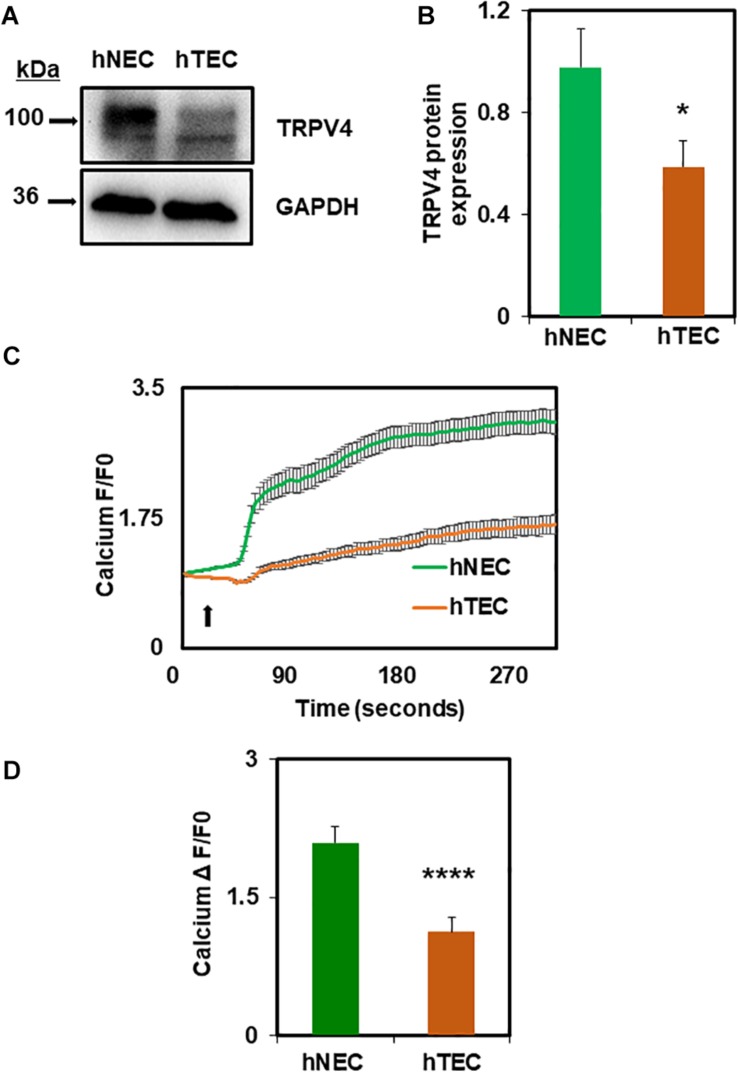
TCM exposure downregulates functional expression of TRPV4 channels in hTEC. **(A)** Western blot analysis showing decreased expression of TRPV4 protein in hTEC when compared to hNEC. **(B)** Quantitative analysis of western blots revealed significant (^∗^*p* ≤ 0.05) reduction of TRPV4 expression in hTEC. **(C)** Average traces showing calcium influx in response to the TRPV4 agonist, GSK1016790A (100 nM), in Fluo-4 loaded hNEC and hTEC. Arrow indicates the time of stimulation with TRPV4 agonist. **(D)** Quantitative analysis of calcium influx showed significant ^****^p ≤ 0.0001 reduction in TRPV4-mediated calcium influx in hTEC compared to hNEC (F/F0 = ratio of normalized fluorescent intensity relative to time 0). The results shown are a mean ± SEM from three independent experiments.

**FIGURE 3:**
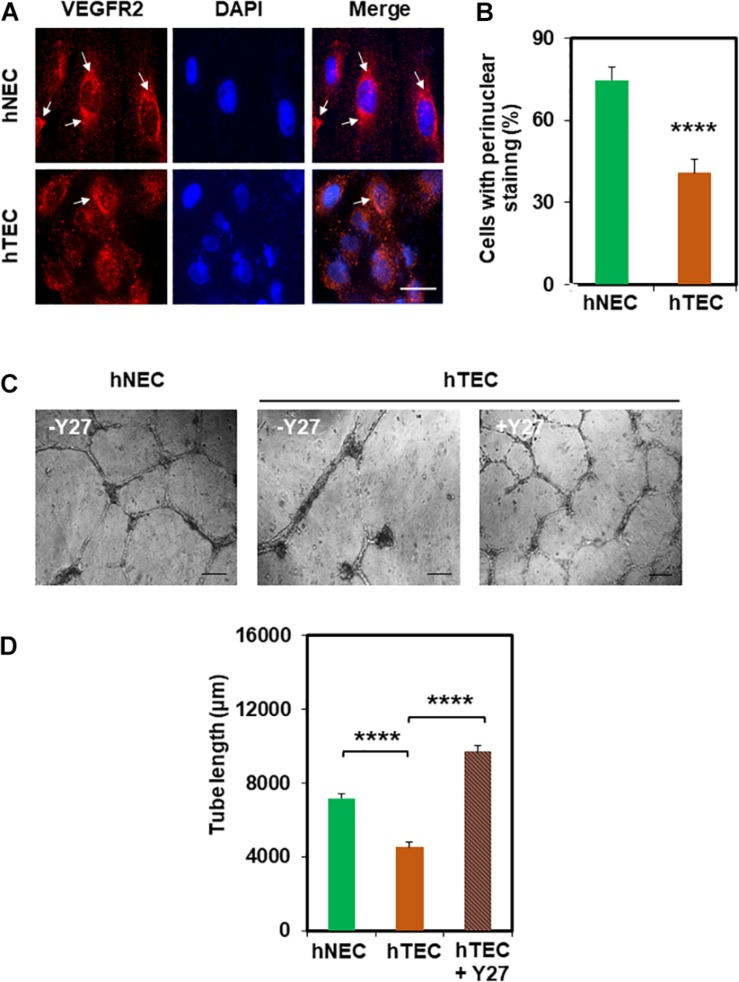
hNEC transformation is correlated with the reduction in perinuclear VEGFR2 which is normalized by Rho kinase inhibitor Y27632. **(A)** Representative immunofluorescence images (60×) showing VEGFR2 localization in hNEC and hTEC. Cells were stained for DAPI (blue/nuclei) and total VEGFR2 (red) after exposure to TCM in 9 consecutive passages (hTEC). Scale bar = 10 μm. Note a strong perinuclear localization of VEGFR2 (arrows) in hNEC which was reduced in hTEC. **(B)** The graph represents the quantitative analysis of the percentage of cells with perinuclear localization of VEGFR2 showing a significant (^****^p ≤ 0.0001) decrease in perinuclear VEGFR2 staining of hTEC. **(C)** Phase contrast micrographs (4×) showing the normalizing effects of the Rho kinase inhibitor, Y-27632 (10 μM; Y27) on hTEC angiogenic behavior, when plated on Matrigel at high density (100,000/well). Representative images were taken 24 h post plating, showing the formation of stable tubes upon inhibition of Rho kinase in hTEC. Scale bar = 200 μm. **(D)** Quantitative analysis of tube length showing a significant decrease (^****^p ≤ 0.0001) in hTEC tube length compared with hNEC which was significantly increased (^****^p ≤ 0.0001) after treatment with the Rho kinase inhibitor. The results shown are a mean ± SEM from three independent experiments.

Next, we investigated the molecular mechanism by which TCM induces EC transformation by focusing on TRPV4 channels. We have previously shown that TRPV4 expression and activity is downregulated in mouse TEC (Adapala et al., 2016). Therefore, first, we measured the expression of TRPV4 in hNEC and hTEC. As shown in Figures 2A,B, western blot analysis revealed that TRPV4 expression (two bands above and below 100 kDa) was significantly lower in hTEC compared to hNEC (*p* ≤ 0.05). Further, calcium imaging revealed that TRPV4-mediated calcium influx in response to the specific agonist GSK1016790A, was significantly lower in hTEC (*p* ≤ 0.0001) compared to hNEC (Figures 2C,D). We next asked if the reduction in TRPV4 altered vascular endothelial growth factor receptor 2 (VEGFR2) or Rho/Rho kinase pathways in hTEC, which we have previously shown to be activated in TRPV4 null (TRPV4KO) or TRPV4 siRNA knocked down EC (Kanugula et al., 2019). Indeed, immunostaining revealed a significant reduction of perinuclear VEGFR2 levels in hTEC compared to hNEC (*p* ≤ 0.0001) (Figures 3A,B). Finally, we found that pre-treatment with Rho kinase inhibitor, Y-27632 (Y27), normalized abnormal angiogenesis phenotype exhibited by hTEC (Figures 3C,D; *p* ≤ 0.0001).

### Extracellular Vesicles From Tumor Cell Conditioned Media Induce Abnormal Angiogenic Phenotype in hNEC

**FIGURE 4:**
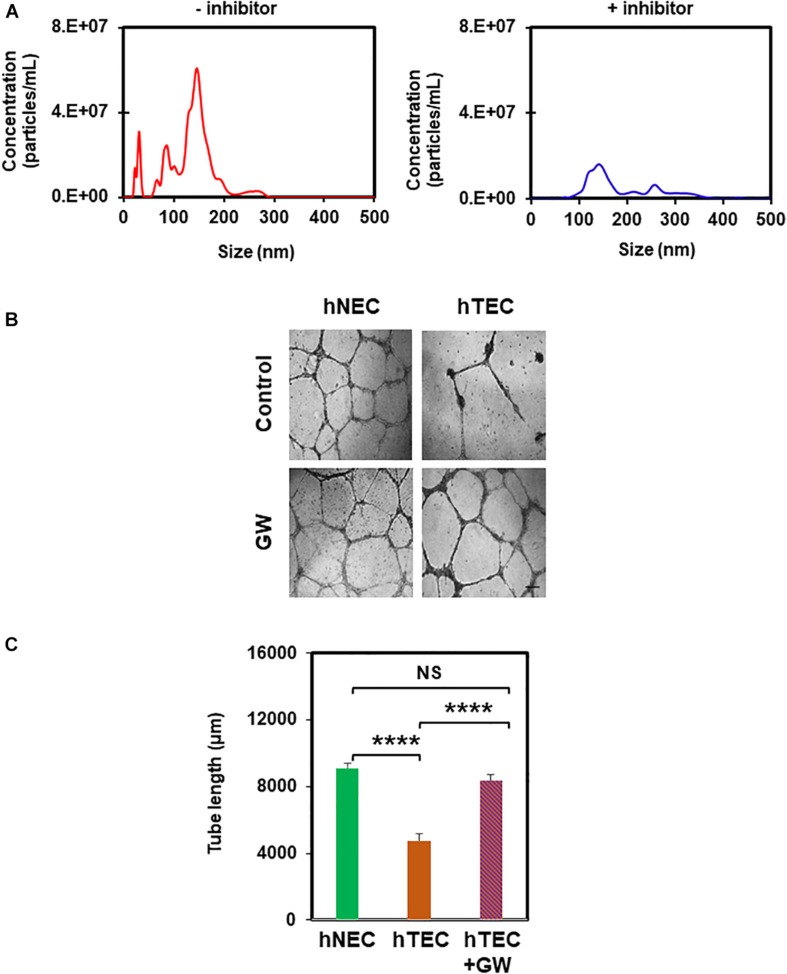
Exosome inhibitor reduces formation of EVs and abolishes TCM-induced EC transformation. **(A)** Nanoparticle tracking analysis (NTA) showing reduction in the number of EVs from tumor cells that had been pre-treated with the exosome inhibitor GW4869 (GW). **(B)** Phase contrast micrographs (4×) showing normalized tube formation in hTEC exposed to GW4869 and plated on 2D Matrigels for 24 h compared to untreated hTEC and hNEC. Sale bar = 200 μm. **(C)** Quantitative analysis showing a significant increase (^****^p ≤ 0.0001) in tube length between hTEC and hTEC + GW (GW4869) cells. Note a significant decrease in tube formation between hNEC and hTEC (^****^p ≤ 0.0001), and no statistical difference (NS) between hNEC and hTEC + GW. The results shown are a mean ± SEM from three independent experiments.

**FIGURE 5:**
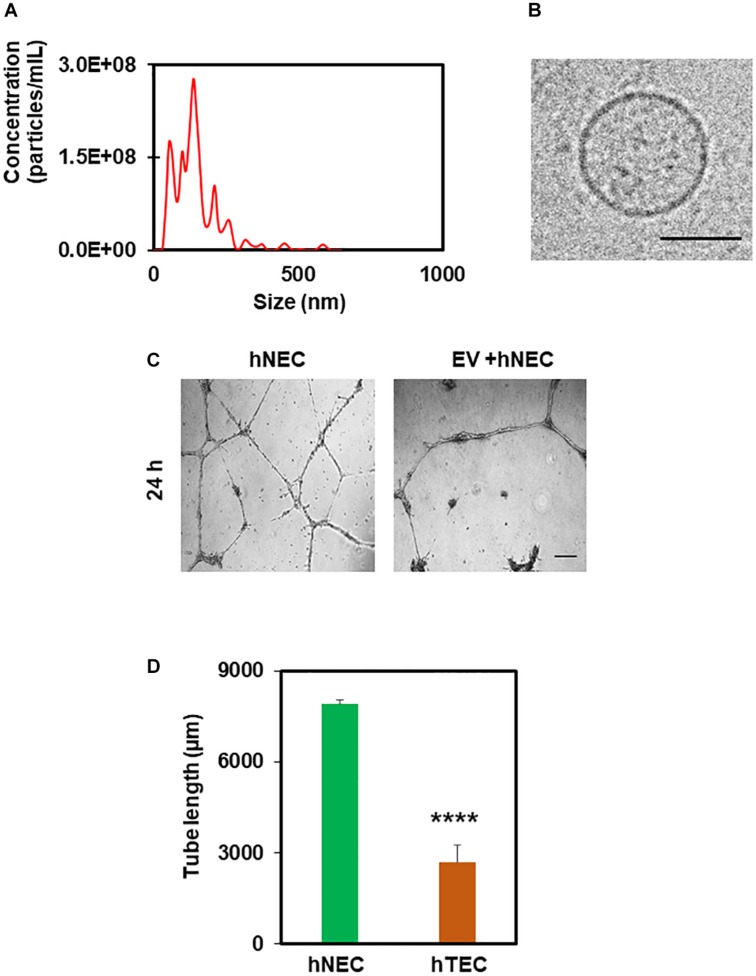
Tumor-derived extracellular vesicles (EVs) induce hNEC transformation to hTEC as evidenced by abnormal angiogenesis. NTA analysis **(A)** and TEM images **(B)** showing 50–200 nm rounded structures confirming the isolation EVs from TCM. Scale bar = 50 nm. **(C)** Phase contrast micrographs (4×) showing tube formation of control and EV-treated cells plated on 2D Matrigel at 24 h. Scale bar = 200 μm. **(D)** Quantitative analysis showing a significant decrease (^****^p ≤ 0.0001) in tube length by EC (hTEC) treated with tumor-derived EVs for 48 h. The results shown are a mean ± SEM from three independent experiments.

In order to find out the active component of TCM that imparts a tumor endothelial-like phenotype, we focused on EVs from TME, which were shown to modulate angiogenesis (Kikuchi et al., 2019). To achieve this, we first treated tumor cells with the exosome inhibitor, GW4869, and measured EV formation using NTA (Nanoparticle Tracking Analysis). As shown in Figure 4A, we found 50–200 nm sized particles in untreated TCM, the levels of which decreased in TCM-treated with GW4869 (Figure 4A). We then exposed hNEC to TCM untreated or treated with GW4869 for 5 consecutive passages and assessed their functional phenotype in a 2D-angiogenesis assay. We found that while cells exposed to untreated TCM formed tubes that collapsed at 24 h, GW4869-TCM exposed cells formed tubes that were stable even at 24 h (Figures 4B,C). Together, these data confirm the presence of EVs in TCM, and that inhibition of exosomes abolishes EC transforming ability of TCM. To further confirm the role of EVs in EC transformation, we isolated EVs from TCM and confirmed their identity with NTA and TEM. While we found 50–200 nm particles in NTA, TEM images showed round structures of 50–150 nm in EVs isolated from TCM (Figures 5A,B). Finally, hNECs treated with purified EVs but not PBS-treated controls, exhibited tumor EC-like tube formation with complete tube retraction at 24 h (*p* ≤ 0.0001) (Figures 5C,D). Taken together, these data confirm that treatment with tumor derived EVs transforms normal EC to tumor endothelial cell-like EC.

## Discussion

The TME is a heterogenous mix of cellular and acellular components including stromal cells, extracellular matrix, soluble factors and EVs secreted by both tumor and stromal cells. Emerging evidence has shown that tumor-derived EVs play an important role in cell-to-cell communication within the TME (Han et al., 2019; Qiao et al., 2019; Tai et al., 2019). EVs have been demonstrated to carry a cargo of DNAs, RNAs, miRNAs, proteins, lipids, and cytokines that facilitate tumor growth, progression and metastasis by delivering them to stromal cells in TME. Specifically, EVs isolated from tumors/tumor cells were shown to induce angiogenesis via interaction with endothelial cells *in vitro* and *in vivo* (Becker et al., 2016; Feng et al., 2017). However, there are no reports on EV’s effects on transformation of normal EC to a tumor EC-like phenotype. In the present study, we demonstrate that repeated exposure to TCM (i.e., pathological tumor microenvironment) transforms normal endothelial cells to a tumor endothelial-like phenotype as evidenced by increased TEM8 expression and abnormal angiogenesis *in vitro*. Further, we show that TCM treatment downregulated functional expression of TRPV4 in EC. Furthermore, we demonstrate that TCM treated with an exosome inhibitor significantly reduced EV production as well as transformation of hNEC. Finally, we found that EVs isolated from TCM induced a tumor endothelial cell-like phenotype.

Although tumors recruit EC from either surrounding vasculature or bone marrow for the initiation of tumor angiogenesis, over time these tumor derived EC (TEC) transform into an aberrant phenotype characterized by increased expression of TEMs, enhanced proliferation, migration and abnormal angiogenesis. However, the molecular mechanisms by which tumors impart this phenotype to TEC is not known. In the present study, we found that exposure to TCM transformed hNEC to hTEC as evidenced by increased expression of TEC marker TEM-8 and abnormal angiogenesis. Importantly, we found that the active component of TCM is EVs as evidenced by (a) their typical round structure of 50–200 nm size confirmed by TEM and NTA, (b) attenuated synthesis and abolishment of transformative effect on hNEC after the treatment with an exosome inhibitor, GW4869, and (c) transformation of hNEC to hTEC-like phenotype by isolated EVs. EVs secreted by tumors increase angiogenesis by delivering pro-angiogenic growth factors or inducing pro-angiogenic genes to endothelial cells (Becker et al., 2016; Feng et al., 2017). EVs derived from glioblastoma and grown in specifically from hypoxic environment, carry number of proangiogenic cytokines and growth factors and stimulate angiogenesis via modulation of HIF, MMP 9 and LOX (King et al., 2012; Kucharzewska et al., 2013). We have previously shown that the abnormal phenotype of TEC, at least in part, comes from the functional downregulation of TRPV4 channels, as overexpression or pharmacological activation of TRPV4 normalizes TEC phenotype (Adapala et al., 2016). Here, we found that exposure to TCM significantly downregulated expression of TRPV4 in hTEC compared to hNEC. Further, TRPV4-mediated calcium influx was significantly attenuated in these cells. Downregulation of TRPV4 has been shown to activate VEGF/VEGFR2 pathway via Rho kinase/YAP (Kanugula et al., 2019), and indeed, we found that TCM treatment significantly reduced perinuclear VEGFR2 suggesting activation of VEGFR2 in hTEC (Manickam et al., 2011; Wang et al., 2017). Further, pharmacological inhibition of Rho kinase was able to normalize abnormal angiogenesis exhibited by hTEC. These findings suggest that TRPV4 downregulation by TCM/EVs transforms hNEC to hTEC via activation of VEGF/VEGFR2 pathway. Although we provide evidence for TRPV4 downregulation by EVs, the underlying molecular mechanism is not known. We speculate that EVs mediate these effects through microRNA, specifically miR-203. miR-203 is the only miRNA shown to target and downregulate TRPV4 expression in chondrocytes and hepatic stellate cells (Hu et al., 2013; Song et al., 2014). However, the role of miR-203 in EV-mediated downregulation of TRPV4 in EC needs to be determined. EVs released into TME either by tumor cells or stromal cells were shown to induce tumor angiogenesis, ECM remodeling and metastasis, albeit through activating known pro-angiogenic signaling molecules such as HIF, MMP, PI3K/AKT and LOX (King et al., 2012; Kucharzewska et al., 2013). However, the findings from our study, for the first time, show that tumor-derived EVs target a mechanosensitive calcium channel, TRPV4 and transforms normal EC to tumor EC-like phenotype, which may explain the mechanisms underlying the abnormal vasculature in the tumor.

## Conclusion

Overall, our results demonstrate that tumor cells communicate with endothelial cells via secretion of EVs into TME, which transforms normal EC into tumor-like EC. These findings have pathophysiological significance as anti-angiogenic therapies based on VEGF have shown limited success in treating cancer (Lupo et al., 2016; Zarrin et al., 2017; Abdalla et al., 2018; Itatani et al., 2018) because tumor cells develop resistance over the time. Moreover, exposure to anti-VEGF molecules was shown to affect tumor perfusion and can impede delivery of chemotherapeutic drugs (Van Der Veldt et al., 2012). Therefore, vascular normalization approaches aimed at restoring tumor vasculature, allowing for effective delivery of chemotherapies (Jain, 2005b; Carmeliet and Jain, 2011; Goel et al., 2011) gained much attention. Our findings show that EVs from tumor cells transform normal endothelial cells to a tumor endothelial cell-like phenotype via downregulation of TRPV4 and identifies TRPV4 as an alternative target for tumor angiogenesis and cancer therapy.

## Data Availability Statement

The datasets generated for this study are available on request to the corresponding author.

## Author Contributions

BG, RA, AK, NL, and JD performed the research, analyzed the data, and edited the manuscript. SP edited the manuscript. MK designed and analyzed the data on exosomes and edited the manuscript. CT designed, interpreted, and analyzed the data as well as wrote the manuscript.

## Conflict of Interest

The authors declare that the research was conducted in the absence of any commercial or financial relationships that could be construed as a potential conflict of interest.
